# Non-invasive convective head cooling during stroke thrombectomy: A prospective multi-center feasibility trial

**DOI:** 10.1093/esj/23969873251371001

**Published:** 2026-01-01

**Authors:** William K Diprose, Catherine Veilleux, Mohammed Almekhlafi, Alec Beresford, Kaustubha Ghate, Davina McAllister, Michael T M Wang, Jessica Wiles, Douglas Campbell, P Alan Barber

**Affiliations:** Department of Medicine, Faculty of Medical and Health Sciences, The University of Auckland, Auckland, New Zealand; Department of Neurology, John Hunter Hospital, Hunter New England Local Health District, New Lambton Heights, NSW, Australia; Department of Clinical Neurosciences, Cumming School of Medicine, University of Calgary, Calgary, AB, Canada; Department of Clinical Neurosciences, Cumming School of Medicine, University of Calgary, Calgary, AB, Canada; Department of Anaesthesia and Perioperative Medicine, Auckland City Hospital, Auckland, New Zealand; Department of Radiology, Auckland City Hospital, Auckland, New Zealand; Department of Anaesthesia and Perioperative Medicine, Auckland City Hospital, Auckland, New Zealand; Department of Medicine, Faculty of Medical and Health Sciences, The University of Auckland, Auckland, New Zealand; Department of Anaesthesia and Perioperative Medicine, Auckland City Hospital, Auckland, New Zealand; Department of Anaesthesia and Perioperative Medicine, Auckland City Hospital, Auckland, New Zealand; Department of Medicine, Faculty of Medical and Health Sciences, The University of Auckland, Auckland, New Zealand; Department of Neurology, Auckland City Hospital, Auckland, New Zealand

**Keywords:** Non-invasive, head cooling, ischemic stroke, neuroprotection

## Abstract

**Introduction:**

Non-invasive convective head cooling is a promising putative neuroprotective therapy for ischemic stroke patients as it may portably, non-invasively, and selectively cool the ischemic penumbra. We aimed to investigate the feasibility of utilizing non-invasive convective head cooling in ischemic stroke patients before and during endovascular thrombectomy (EVT).

**Patients and methods:**

We conducted a multi-center, prospective, non-randomized, open-label trial at two comprehensive stroke centers in ischemic stroke patients where EVT was planned. Patients were assessed for eligibility in the emergency department (ED) and had a cooling cap fitted that circulated coolant between −5°C and 0°C until EVT completion. The primary feasibility endpoint was adherence, defined as tolerating cooling for ⩾50% of the time from cooling cap application until EVT completion.

**Results:**

Between July and November 2024, 40 EVT patients (19 (47.5%) female, mean ± SD age 71.6 ± 12.6 years) underwent a median (IQR) duration of convective head cooling of 86 (58–106) min. Thirty-nine (97.5%) participants met the primary feasibility endpoint. The enrollment rate was five participants per site per month. Median (IQR) time from comprehensive stroke center arrival to cooling start was 10 (5–51) min. Thirty-two (80%) patients received general anesthesia. eTICI 2b-3 reperfusion was achieved in 38 (95.0%) participants. Median (IQR) 24-h infarct volume was 14.3 (5.5–29.1) mL. Median (IQR) 3-month modified Rankin Scale score was 2 (1–5). Three-month mortality occurred in 8/38 (21.1%) participants. Nine serious adverse events occurred in 8 (20.0%) participants, none of which were attributed to head cooling.

**Conclusions:**

Convective head cooling is feasible in patients undergoing EVT and warrants further investigation in larger randomized controlled trials.

## Graphical Abstract

**Graphical abstract:**
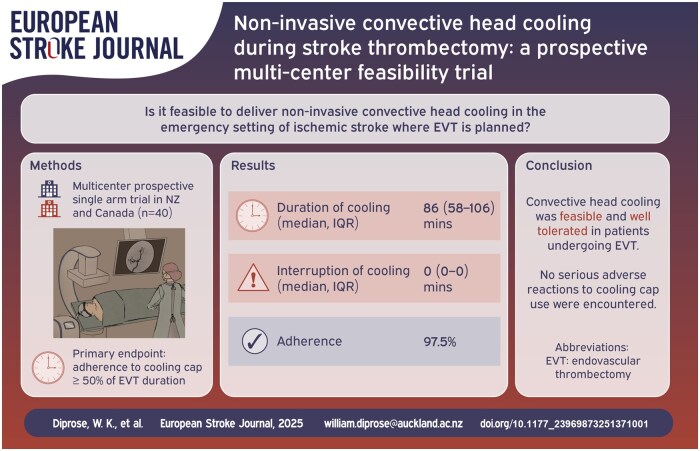

## Introduction

There is renewed interest in therapies aimed at slowing the expansion of the infarct core into the ischemic penumbra prior to reperfusion in ischemic stroke.^1^ Therapeutic hypothermia is a widely researched neuroprotectant in animal models of ischemic stroke,^2–4^ and is an established treatment for both neonatal and post-cardiac arrest hypoxic ischemic encephalopathy.^5–7^ During cerebral ischemia, lower brain temperatures reduce metabolic demand, release of excitatory neurotransmitters, free-radical production and breakdown of the blood-brain barrier, thereby reducing cell death and infarct volume.^8^ Meta-analyses of preclinical studies have consistently shown that hypothermia reduces infarct size by ~44% in animal models of focal ischemia.^3,4^ The effect size of hypothermia is greatest in animal models of temporary focal ischemia, that is, animals with reperfusion.^3^,^4^

Clinical trials have so far failed to demonstrate the benefit of therapeutic hypothermia in humans with ischemic stroke.^9,10^ Most human trials have focused on inducing systemic hypothermia through body surface or intravenous cooling, both of which are practically challenging and can lead to significant systemic complications such as pneumonia.^9,10^ Moreover, trials have not specifically focused on patients with a high likelihood of achieving reperfusion, such as those undergoing endovascular thrombectomy (EVT).^11^ Convective (“active conductive”) head cooling can non-invasively and selectively reduce brain temperature,^12–16^ and could potentially be applied in the pre-hospital setting, the inter-hospital transfer, and throughout the EVT procedure, but it has not yet been tested.^16,17^

## Aims

We conducted a multi-center, prospective, non-randomized, open-label trial. Our major aim was to assess feasibility of delivery of non-invasive convective head cooling in the emergency setting of ischemic stroke where EVT is planned.

## Materials and methods

### Study population and intervention

This study is reported according to the Consolidated Standards of Reporting Trials (CONSORT) guidelines.^18^ The data that support the findings of this study are available from the corresponding author upon reasonable request. The study was conducted at two comprehensive stroke centers (CSCs) in New Zealand and Canada. The study was prospectively registered with the Australian New Zealand Clinical Trials Registry (ACTRN12621001346864p) and ClinicalTrials.gov (NCT06335641).

Consecutive patients with anterior circulation ischemic stroke where EVT was planned were considered eligible for enrollment. Exclusion criteria were admission core body temperature <35°C; blood pressure ⩾185/110 mmHg not responsive to guideline-directed intravenous antihypertensive therapy; known contraindications to hypothermia including hemodynamically unstable patients, new or symptomatic bradyarrhythmia, hematologic dyscrasias that affect thrombosis, or vasospastic disorders such as Raynaud’s syndrome or thrombo-angiitis obliterans; skin lesions not allowing secure application of the cooling cap; or usual residence outside of New Zealand or Canada. Intravenous thrombolysis was not an exclusion criterion. In New Zealand, a “best interest agreement” was used to enroll participants, whereby an independent physician and a study investigator both agreed that enrollment in the trial was in the patient’s best interest in accordance with New Zealand law and right 7(4) of the Health and Disability Commissioner code. In Canada, the consenting process was to seek informed consent from the patient or surrogate, and if none were available, then two-physician consent was obtained. Participants could elect to continue or to withdraw from the study if they regained the ability to consent. An independent Data Safety Monitoring Committee met after the enrollment of 20 participants to review trial progress and any serious adverse events.

Demographic characteristics, medical history, and stroke symptoms and severity were assessed at presentation. Baseline physiological data were collected by the study team, including body temperature (tympanic), blood pressure, and heart rate before initiation of head cooling. Head cooling was achieved with either the WElkins Temperature Regulation System, 2nd Gen (TRS-2), or the Orbis Paxman System (Paxman Coolers Ltd, Huddersfield, Great Britain). The TRS-2 consists of a cooling unit which circulates coolant through umbilical tubing into a cooling cap. The cooling cap contacts the participant’s scalp, and depending on the model of cap used, the posterolateral neck, with delivered temperature ranging between −5°C and 0°C in this study. WElkins cooling systems have been previously trialed in several neurological conditions, including ischemic stroke and traumatic brain injury.^12,13^ The Orbis Paxman System is a free-standing, electrically powered, mobile refrigeration unit which circulates coolant at −4°C through a cooling cap that is attached to, and covers, the top of the participant’s head. The Orbis Paxman system is intended by the manufacturer to be used for scalp cooling of patients who are receiving chemotherapy to reduce the risk of chemotherapy-induced alopecia.

Head cooling was commenced prior to EVT in the emergency department or angiography suite of the CSC and continued until either the end of the procedure or until 120 min of cooling had been completed, even if the procedure was still underway. The duration of cooling was selected with the goal of demonstrating feasibility, informed by our previous pilot study COOLHEAD-1,^16^ where the tolerability of convective head cooling was demonstrated in awake volunteers for 120 min. Physiological monitoring continued during head cooling as part of routine clinical care, including either intermittent non-invasive or continuous invasive arterial blood pressure, heart rate, oximetry, and nasopharyngeal temperature if general anesthesia was performed. The choice between local anesthesia, conscious sedation and general anesthesia was at the discretion of the neurointerventionalist and neuroanesthesiologist.

Pre-specified criteria for discontinuing head cooling included the following: if a member of the clinical team felt that the participant was not tolerating head cooling; cold-related shivering for >5 min despite the administration of passive systemic warming; body temperature reached ⩽35°C; blood pressure above 185/110 mmHg or an absolute increase in systolic blood pressure of 30 mmHg from baseline on two consecutive measurements, that was resistant to standard pharmacologic antihypertensive treatment; or if there was an unexpected anesthesia or medical event (e.g. symptomatic bradycardia), or procedural complication (e.g. vessel dissection or intracerebral hemorrhage). A post-cooling body temperature measurement was performed in the post-anesthesia care unit, stroke unit, or intensive care unit, depending on the patient’s disposition. Post-procedure care was in accordance with the hospital protocols.

### Outcomes

The primary feasibility outcome was the proportion of participants who were adherent to the intervention. Adherence was defined as undergoing head cooling for ⩾50% of the time from first application of the cooling cap until the end of the EVT procedure, or after 120 min of cooling (whichever came first). Secondary feasibility outcomes included interruption of cooling and the monthly enrollment rate. Process outcomes included CSC arrival to cooling start time, CSC arrival to arterial access time, and arterial access to reperfusion time.

Safety outcomes included symptomatic bradyarrhythmia, uncontrolled hypertension, or cold-related shivering during head cooling; cervical or cerebral vasospasm requiring the administration of intra-arterial vasodilators; symptomatic intracranial hemorrhage, defined as new intracranial hemorrhage with National Institutes of Health Stroke Scale (NIHSS) score decline of ⩾4 points with the hemorrhage judged to be the most important cause of clinical worsening^19^; pneumonia according for the Center for Disease Control criteria within 7 days of stroke onset or discharge (whichever comes first)^20,21^; and mortality by 3 months.

Efficacy outcomes included rate of good reperfusion defined as Expanded Treatment in Cerebral Infarction (eTICI) score of 2b-3; rate of excellent reperfusion defined as eTICI score 2c-3; 24-h infarct volume, performed through manual planimetric measurements on axial non-contrast CT head follow-up imaging at 24 h using the open-source software ITK snap (http://www.itksnap.org); early neurological improvement, defined as a 30% or greater improvement in the NIHSS score at 24 h^22^; excellent functional outcome, defined as a modified Rankin Scale (mRS) score of 0 or 1, at 3 months; and good functional outcome, defined as an mRS of 0, 1, or 2, at 3 months. The mRS score at 3 months was determined using the Rankin Focused Assessment by telephone interview conducted by a trained researcher.

### Statistical analysis

We used the confidence interval (CI) approach to estimate the sample size required to establish feasibility.^23^ In order to be able to estimate an adherence rate of 90% to within a 95% CI of ±10%, a minimum sample size of *n* = 36 was required. To allow for potential loss of follow-up of up to 10%, we planned to enroll a total of *n* = 40 participants. Statistical analysis was performed with R version 4.4.2 (R Foundation). Data are reported using standard descriptive statistics. Continuous variables are expressed as mean (standard deviation (SD)) or median (interquartile range (IQR)), while categorical variables are presented as absolute numbers and percentages.

## Results

**Figure 1.**
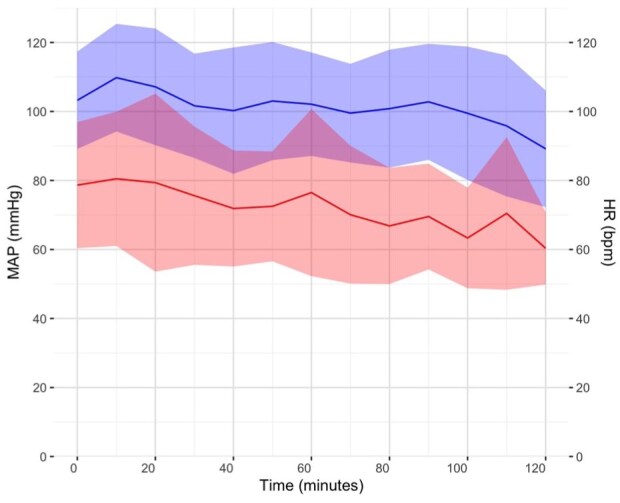
Mean arterial pressure (MAP, mmHg) and heart rate (HR, beats per minute (bpm)) during head cooling. The blue line is MAP and the red line is HR. Colored ribbons represent the standard deviation at each time point.

**Figure 2.**
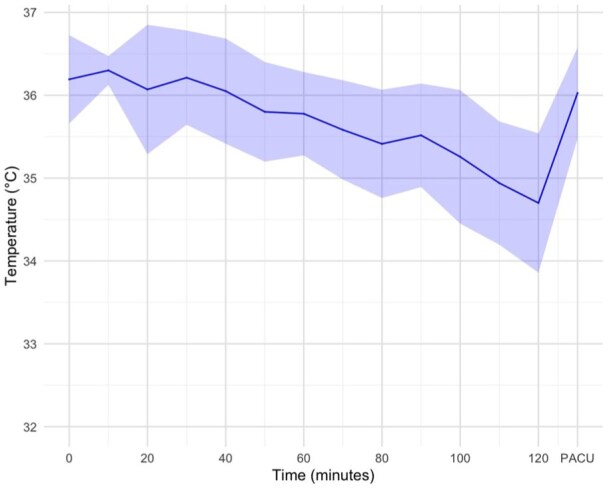
Body temperature in degree Celsius (°C) before, during, and after head cooling. Colored ribbons represent the standard deviation at each time point. PACU, post-anesthesia care unit.

Between July and November 2024, 40 EVT patients (19 (47.5%) female, mean ± SD age 71.6 ± 12.6 years) were prospectively enrolled and underwent non-invasive convective head cooling for a median (IQR) duration of 86 (58–106) min. Baseline characteristics are summarized in Table 1. Median (IQR) last known normal to CSC arrival time was 318 (169–697) min. Median (IQR) NIHSS was 16 (10–20) and median (IQR) Alberta Stroke Program Early CT Score (ASPECTS) was 9 (7–9). Sixteen (40%) participants were treated with intravenous thrombolysis. Thirty-two (80%) of participants received general anesthesia. Physiological parameters before, during, and after head cooling are summarized in Figures 1 and 2.

**Table 1.** Baseline characteristics of head cooling patients.

Demographics (*n* = 40)	
Age, mean (standard deviation), years	71.6 (12.6)
Age, ⩾80 years	13 (32.5)
Female	19 (47.5)
European ethnicity	28 (70)
Clinical	
LKN to CSC, median (IQR), min	318 (169–697)
Medical history	
Previous ischemic stroke/TIA	9 (22.5)
Hypertension	25 (62.5)
Diabetes	4 (10)
Dyslipidemia	19 (47.5)
Atrial fibrillation	17 (42.5)
Current smoker	5 (12.5)
Pre-stroke mRS, median (IQR)	0 (0–0)
Pre-stroke mRS ⩾2	6 (15)
Baseline NIHSS score, median (IQR)	16 (10–20)
ASPECTS, median (IQR)	9 (7–9)
ASPECTS ⩽5	3 (7.5)
Intravenous thrombolysis	16 (40)
Site of occlusion	
ICA	6 (15)
M1-MCA	20 (50)
M2-MCA	10 (25)
Other	3 (7.5)
Stroke etiology	
Cardioembolic	22 (55)
Large artery atherosclerosis	9 (22.5)
Undetermined	9 (22.5)
Anesthetic technique	
General anesthesia	32 (80)
Conscious sedation	8 (20)

ASPECTS: Alberta Stroke Program Early CT Score; CSC: comprehensive stroke center; ICA: internal carotid artery; LKN: last known normal; mRS: modified Rankin Scale; M1-MCA: first segment of the middle cerebral artery; M2-MCA: segment segment of the middle cerebral artery; NIHSS: National Institutes of Health Stroke Scale; TIA: transient ischemic attack.

Feasibility and process outcomes are summarized in Table 2. The primary feasibility outcome was achieved in 39 (97.5%) participants. One participant did not meet the primary feasibility outcome. This patient underwent EVT with local anesthesia, and cooling was stopped because of concerns that the cooling cap could interfere with a head immobilizer (Adept Medical Head Immobilizer) that secures and supports the head with a vacuum suction system during awake neuroangiographic procedures. Median (IQR) cooling interruption was 0 (0–0) min. The enrollment rate was five participants per site per month. Median (IQR) CSC arrival to cooling start time was 10 (5–51) min. Median (IQR) CSC arrival to arterial access time was 45 (32–97) min.

**Table 2.** Feasibility, safety, and efficacy outcomes in head cooling patients.

Feasibility	
Adherence	39 (97.5)
Interruption of cooling, median (IQR), min	0 (0–0)
Enrollment rate	5 participants/site /month
Process	
CSC to cooling start time, median (IQR), min	10 (5–51)
CSC to arterial access, median (IQR), min	45 (32–97)
Arterial access time to reperfusion time, median (IQR), min	28 (17–60)
Safety	
Shivering	7 (17.5)
Cervical or cerebral vasospasm requiring IA vasodilators	5 (12.5)
Procedure-related complications^a^	3 (7.5)
Pneumonia	2 (5.0)
Symptomatic bradyarrhythmia	1 (2.5)
Uncontrolled hypertension	1 (2.5)
Symptomatic ICH^b^	0 (0)
Mortality by 3 months	8/38 (21.1)
Efficacy	
Good reperfusion^c^	38 (95.0)
Excellent reperfusion^d^	32 (80)
24-h infarct volume, mean (SD)	34.0 (60.5)
24-h infarct volume, median (IQR)	14.3 (5.5–29.1)
24-h NIHSS, median (IQR)	6 (2–11)
Early neurological improvement, *n* (%)^e^	28 (70)
Excellent functional outcome, *n* (%)^f^	14/38 (36.8)
Good functional outcome, *n* (%)^g^	20/38 (52.6)

ICH: intracerebral hemorrhage; IA: intra-arterial; CSC: comprehensive stroke center; NIHSS: National Institutes of Health Stroke Scale.

^a^Two groin hematomas and one femoral artery pseudoaneurysm.

^b^Defined as new intracranial hemorrhage with NIHSS score decline of ⩾4 points with the hemorrhage judged to be the most important cause of clinical worsening.

^c^Expanded treatment in cerebral infarction (eTICI) score 2b-3.

^d^eTICI score 2c-3.

^e^Thirty percentage or greater improvement in the National Institutes of Health Stroke Scale (NIHSS) score at 24 h.

^f^Modified Rankin Scale (mRS) score of 0 or 1, at 3 months.

^g^Modified Rankin Scale (mRS) score of 0, 1, or 2 at 3 months.

Safety outcomes are summarized in Table 2. Three-month follow-up was missing for 2/40 (5%) of participants. Nine serious adverse events occurred in eight (20%) participants, none of which were attributed to head cooling by the independent Data Safety Monitoring Committee. Shivering occurred in seven (17.5%) participants, six (85.7%) of whom received general anesthesia. Cervical or cerebral vasospasm requiring the administration of intra-arterial vasodilators occurred in five (12.5%) participants. One (2.5%) participant had uncontrolled hypertension during head cooling and one (2.5%) developed transient bradycardia and hypotension in the post-anesthesia care unit after head cooling had been stopped. Two (5.0%) participants developed pneumonia. Two (5.0%) participants developed a groin hematoma, neither of which required intervention, and one (2.5%) developed a groin pseudoaneurysm that required ultrasound-guided thrombin injection. Eight of 38 (21.1%) participants had died by 3 months, five during hospital admission.

**Figure 3.**
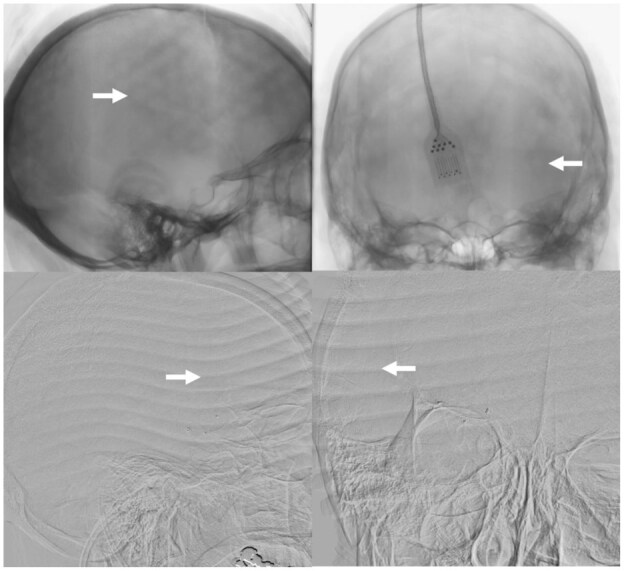
Digital subtraction angiography imaging artifacts caused by the cooling caps. Top panels show “dimpling” artifact from the WElkins Temperature Regulation System, 2nd Gen (TRS-2) cap indicated by white arrows on un-subtracted lateral and anteroposterior projections. Bottom panels show “line” artifact from the Orbis Paxman System cap indicated by white arrows on subtracted lateral and anteroposterior projections.

**Figure 4.**
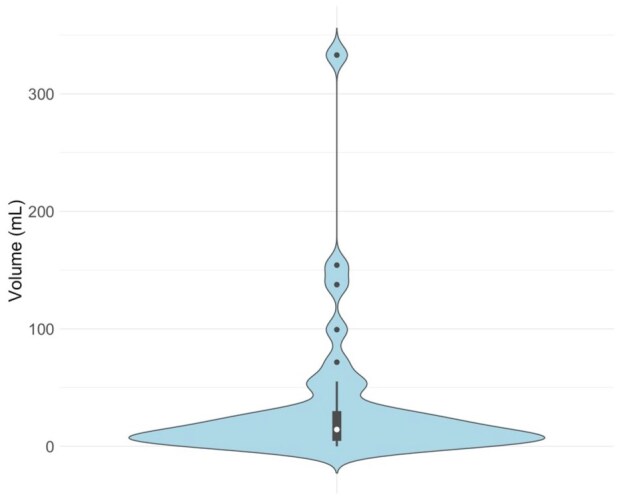
Violin plot depicting 24-h infarct volumes.

**Figure 5.**
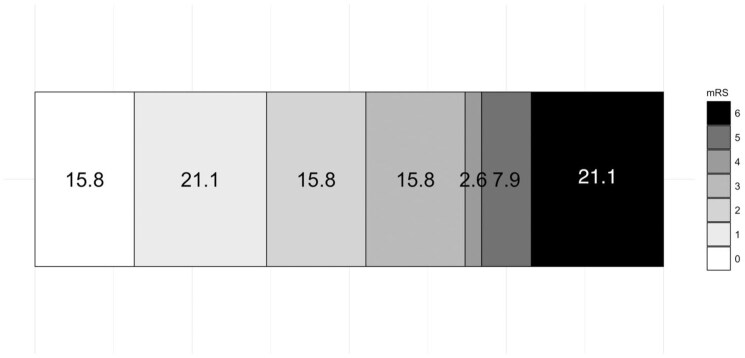
Distribution of 3-month modified Rankin Scale (mRS) scores. Scores on the mRS range from 0 to 6, a 7-point ordered categorical scale with scores ranging from 0 (no disability) to 6 (death). Numbers indicate percent of patients in each mRS category.

Efficacy outcomes are summarized in Table 2. eTICI 2b-3 reperfusion was achieved in 38 (95.0%), and eTICI 2c-3 reperfusion in 32 (80%) of participants. Examples of digital subtraction angiography imaging with the cooling cap in situ are shown in Figure 3. Median (IQR) 24-h NIHSS was 6 (2–11), and median (IQR) percentage 24-h improvement in NIHSS was 63.9% (23.2%–78.3%). Early neurological improvement was achieved in 28 (70%) participants. Mean ± SD and median (IQR) 24-h infarct volumes were 34.0 ± 60.4 mL and 14.3 (5.5–29.1) mL respectively (Figure 4). Median (IQR) mRS at 3 months was 2 (1–5). Excellent functional outcome (mRS 0–1) was achieved in 14/38 (36.8%), and good functional outcome (mRS 0–2) was achieved in 20/36 (52.6%) participants at 3 months (Figure 5).

## Discussion

This multi-center, prospective, non-randomized, open-label trial demonstrated that non-invasive convective head cooling was successfully delivered in the emergency department to ischemic stroke patients in whom EVT was planned. The median duration of head cooling in this study was 86 min. The primary feasibility outcome, adherence to head cooling, was achieved in 97.5% of participants. Secondary feasibility outcomes, including work-flow times, reperfusion rate, and enrollment rate indicated that head cooling was successfully incorporated into the clinical workflow without compromising the current standard of care. Larger randomized controlled trials testing the safety and efficacy of head cooling in ischemic stroke patients prior to reperfusion are warranted.

Clinical trials of therapeutic hypothermia in ischemic stroke have failed largely due to the infeasibility of whole body cooling.^9,10^ ICTuS-2 aimed to randomize 1600 patients treated with IVT to either whole body cooling (target 33^o^C for 24 h via intravenous cooling) or normothermia.^9^ Only 120 patients were enrolled over 55 months at 31 sites (enrollment rate 0.07 patients per site per month), and the trial was terminated early because of the introduction of EVT and the expiration of funding. EuroHYP-1 aimed to randomize 1500 patients to either whole body cooling (target 34.0°C–35.0°C for 12–24 h via either surface or intravenous cooling) or normothermia.^10^ Only 98 patients were enrolled over 60 months at 36 sites (enrollment rate 0.05 patients per site per month), and the trial was stopped early after slow enrollment and cessation of funding. The enrollment rate in the current study (five participants per site per month) was 100 times higher than ICTuS-2 and EuroHYP-1, suggesting that non-invasive convective head cooling may be a more practical alternative to whole body cooling.

Non-invasive head cooling comprises a range of methods that attempt to cool the brain by losing heat through the skull and/or neck by conduction and/or convection, each with their own advantages and disadvantages.^24^ For example, nasopharyngeal cooling whereby heat is lost from the upper airways by convection (with gas or fluid flow) or by conduction (with nasal or pharyngeal balloons) has been shown to reduce brain temperature,^24,25^ but often requires the patient to be sedated and intubated and is not truly non-invasive.^24^ In contrast, “passive” conductive head cooling, with the use of an elasto-gel cranial cap over the head is often well-tolerated, but has not been consistently shown to reduce brain temperature.^24,26^ We and others, have shown that convective (“active conductive”) head cooling with a cooling cap continuously circulating chilled fluid around the scalp and neck, is both well-tolerated in awake patients, and reduces brain temperature using both invasive and non-invasive temperature measurement methods.^12–16^

The earliest description of convective head cooling to selectively reduce brain temperature randomized 14 patients with stroke or traumatic brain injury to receive 48–72 h of head and neck cooling or no cooling.^11^ Brain temperature was continuously monitored via a probe placed 8 mm below the cortical surface and compared with bladder temperature. After 1 h of cooling, brain temperature had reduced by 1.84°C, and after 3.4 h it had fallen below 34°C. The mean brain-bladder temperature difference was −1.6°C in the cooled group and +0.22°C in controls. A subsequent case report provided further evidence of selective brain cooling in a patient with markedly reduced cerebral blood flow due to internal carotid artery occlusion.^14^ The patient received 10 h of convective cooling, and after 1 h the temperature of the ischemic hemisphere had reduced by 8°C.

Qiu et al. evaluated a cooling cap and neckband in 45 patients with traumatic brain injury, compared to 45 non-cooled controls.^14^ Brain temperature was measured 10 mm below the cortical surface, alongside core (rectal) temperature. Selective brain cooling was achieved, with brain temperature maintained at ~35°C while core temperature remained at ~37°C. Another randomized trial involving 25 patients with traumatic brain injury demonstrated that convective head and neck cooling led to a 1.1°C reduction in invasively measured brain temperature. More recently, our pilot study COOLHEAD-1 investigated the temperature-lowering effects of convective head cooling in 11 healthy volunteers using the Welkins cooling device.^16^ Brain temperature was assessed non-invasively with magnetic resonance spectroscopic imaging. After 80 min of cooling, there was a significant reduction in whole-brain temperature (Δ*T* = −0.9 ± 0.7°C, *p* = 0.002), and a smaller but statistically significant reduction in rectal temperature (Δ*T* = −0.3 ± 0.1°C, *p* = 0.03).

In the current study, non-invasive convective head cooling had an acceptable safety profile. Theoretically, the slight radio-opacity of the cooling cap could degrade image quality during EVT and hinder efforts to safely recanalize the occluded vessel. However, eTICI 2c-3 and eTICI 2b-3 reperfusion rates were achieved in 80% and 95% of study participants respectively, higher than was reported in contemporary EVT trials (eTICI 2b-3–80%).^27^ Cervical or cerebral vasospasm requiring the administration of intra-arterial vasodilators was observed in 12.5% of study participants, which is higher than the rate of vasospasm requiring the administration of nimodipine in the ETIS registry of 3.2%,^28^ but within the range of 3.9%–23% reported in randomized controlled trials.^29^ Cooling was stopped in one participant because of concerns that the cooling cap could interfere with a head immobilizer (Adept Medical Head Immobilizer); however, subsequent testing demonstrated satisfactory compatibility between the cooling cap and the head immobilizer.

Shivering occurred in 17.5% of study participants, but this mostly occurred following general anesthesia, where shivering is a common side effect reported in 5%–65% of patients.^30^ Pneumonia within 7 days occurred in 5% of study participants. We used the same definition as ICTuS-2, which reported pneumonia in 10.5% of normothermia and 19% of hypothermia patients.^9^ An observational study of anterior circulation large vessel occlusion stroke patients reported that pneumonia occurs in 19.4% of patients, but this study included pneumonia that developed beyond 7 days.^31^ The median infarct volume at 24 h in our study compared favorably to the active (nerenetide) arm of the contemporary neuroprotection trial, ESCAPE-NEXT (14 vs 43 mL), as did the rates of excellent (37% vs 31%) and good (53% vs 45%) functional outcomes.^32^ Mortality by 3 months occurred in 21% of participants with follow up data, which is comparable to the rate of 19% reported in ESCAPE-NEXT.^31^

There was an overall trend of decreasing body temperature, mean arterial pressure, and heart rate during head cooling. However, 80% of study participants received general anesthesia, in many cases soon after commencing head cooling. Anesthetic drugs may impair normal thermoregulatory responses, cause peripheral vasodilation, and have negative chronotropic effects, leading to the physiological changes seen in the current study.^33^ An earlier study in awake volunteers measured axillary or rectal temperatures, blood pressure, and heart rate during up to 2 h of head cooling, and demonstrated no significant changes in these measures over time.^16^ In contrast, a study of patients with severe ischemic or hemorrhagic stroke reported an average increase in mean arterial pressure of ~7 mmHg during head cooling.^26^

Body temperature measurements were not standardized in the current study. Typically, baseline measurements were performed with a tympanic thermometer in the emergency department, intra-procedural measurements were performed with a naso- or oropharyngeal probe during general anesthesia, and with a sublingual thermometer in the post-anesthesia care unit. The pattern of temperature changes observed in the current study could therefore reflect (1) randomness; (2) impaired thermoregulation secondary to general anesthesia; (3) systemic cooling caused by head cooling; or (4) differences in measurement methods, for example, naso- and oropharyngeal temperature measurements could indicate regional, and not core body temperatures during head cooling.

The current study has limitations. Patients were enrolled at a CSC, and therefore the feasibility of head cooling at primary stroke centers or during interhospital transfer remains unknown. The majority of patients received general anesthesia soon after commencing head cooling, and the maximum duration of head cooling was 120 min. We acknowledge that based on pre-clinical evidence, longer cooling durations may be needed if efficacy were to be investigated in future trials. There was no comparator group, so the influence of head cooling on outcomes is unknown. Brain temperature was not measured, therefore the impact of head cooling on brain-body temperature differential could not be determined. Infarct volumetry and outcome assessments were not blinded, potentially leading to bias. A major strength of the current study were the inclusive eligibility criteria, resulting in approximately one-third of participants being 80 years of age or older, 15% having pre-stroke disability, and 7.5% with large ischemic strokes at presentation.

In summary non-invasive convective head cooling can be successfully delivered in the emergency setting of ischemic stroke where EVT is planned, and warrants further investigation in larger randomized controlled trials.

## Trial registration

The study was prospectively registered with the Australian New Zealand Clinical Trials Registry (ACTRN12621001346864p) and ClinicalTrials.gov (NCT06335641).

## References

[1] Lyden PD. Cerebroprotection for acute ischemic stroke: looking ahead. Stroke 2021; 52: 3033–3044. 34289710 10.1161/STROKEAHA.121.032241 PMC8384682

[2] van der Worp HB, Macleod MR, Kollmar R. Therapeutic hypothermia for acute ischemic stroke: ready to start large randomized trials? J Cereb Blood Flow Metab 2010; 30: 1079–1093. 20354545 10.1038/jcbfm.2010.44 PMC2949207

[3] van der Worp HB, Sena ES, Donnan GA, et al. Hypothermia in animal models of acute ischaemic stroke: a systematic review and meta-analysis. Brain 2007; 130: 3063–3074. 17478443 10.1093/brain/awm083

[4] Dumitrascu OM, Lamb J, Lyden PD. Still cooling after all these years: meta-analysis of pre-clinical trials of therapeutic hypothermia for acute ischemic stroke. J Cereb Blood Flow Metab 2016; 36: 1157–1164. 27089911 10.1177/0271678X16645112 PMC4929706

[5] Gluckman PD, Wyatt JS, Azzopardi D, et al. Selective head cooling with mild systemic hypothermia after neonatal encephalopathy: multicentre randomised trial. Lancet 2005; 365: 663–670. 15721471 10.1016/S0140-6736(05)17946-X

[6] Abate BB, Bimerew M, Gebremichael B, et al. Effects of therapeutic hypothermia on death among asphyxiated neonates with hypoxic-ischemic encephalopathy: a systematic review and meta-analysis of randomized control trials. PLoS One 2021; 16: e0247229. 10.1371/journal.pone.0247229 PMC7906350 33630892

[7] Chiu P-Y, Chung C-C, Tu YK, et al. Therapeutic hypothermia in patients after cardiac arrest: a systematic review and meta-analysis of randomized controlled trials. Am J Emerg Med 2023; 71: 182–189. 37421815 10.1016/j.ajem.2023.06.040

[8] Ginsberg MD, Busto R. Combating hyperthermia in acute stroke: a significant clinical concern. Stroke 1998; 29: 529–534. 9472901 10.1161/01.str.29.2.529

[9] Lyden P, Hemmen T, Grotta J, et al. Results of the ICTuS 2 trial (Intravascular cooling in the treatment of stroke 2). Stroke 2016; 47: 2888–2895. 27834742 10.1161/STROKEAHA.116.014200 PMC5134910

[10] van der Worp HB, Macleod MR, Bath PM, et al. Therapeutic hypothermia for acute ischaemic stroke. Results of a European multicentre, randomised, phase III clinical trial. Eur Stroke J 2019; 4: 254–262. 31984233 10.1177/2396987319844690 PMC6960691

[11] Diprose WK, Liem B, Wang MTM, et al. Impact of body temperature before and after endovascular thrombectomy for large vessel occlusion stroke. Stroke 2020; 51: 1218–1225. 32102631 10.1161/STROKEAHA.119.028160

[12] Wang H, Olivero W, Lanzino G, et al. Rapid and selective cerebral hypothermia achieved using a cooling helmet. J Neurosurg 2004; 100: 272–277. 15086235 10.3171/jns.2004.100.2.0272

[13] Harris OA, Muh CR, Surles MC, et al. Discrete cerebral hypothermia in the management of traumatic brain injury: a randomized controlled trial. J Neurosurg 2009; 110: 1256–1264. 19249933 10.3171/2009.1.JNS081320

[14] Qiu W, Shen H, Zhang Y, et al. Noninvasive selective brain cooling by head and neck cooling is protective in severe traumatic brain injury. J Clin Neurosci 2006; 13: 995–1000. 17113984 10.1016/j.jocn.2006.02.027

[15] Wang H, Wang D, Lanzino G, et al. Differential interhemispheric cooling and ICP compartmentalization in a patient with left ICA occlusion. Acta Neurochir 2006; 148: 681–683. 16502336 10.1007/s00701-006-0748-y

[16] Diprose WK, Morgan CA, Wang MT, et al. Active conductive head cooling of normal and infarcted brain: a magnetic resonance spectroscopy imaging study. J Cereb Blood Flow Metab 2022; 42: 2058–2065. 35707879 10.1177/0271678X221107988 PMC9580175

[17] Diprose WK, Rao A, Ghate K, et al. Penumbral cooling in ischemic stroke with intraarterial, intravenous or active conductive head cooling: a thermal modeling study. J Cereb Blood Flow Metab 2024; 44: 66–76. 37734834 10.1177/0271678X231203025 PMC10905634

[18] Hopewell S, Chan A-W, Collins GS, et al. CONSORT 2025 statement: updated guideline for reporting randomised trials. BMJ 2025; 389: e081123. 10.1136/bmj-2024-081123 PMC11995449 40228833

[19] von Kummer R, Broderick JP, Campbell BC, et al. The Heidelberg bleeding classification: classification of bleeding events after ischemic stroke and reperfusion therapy. Stroke 2015; 46: 2981–2986. 26330447 10.1161/STROKEAHA.115.010049

[20] National Healthcare Safety Network. Pneumonia (Ventilator-associated [VAP] and non-ventilator-associated Pneumonia [PNEU]) Event. https://www.cdc.gov/nhsn/pdfs/pscmanual/6pscvapcurrent.pdf (2025).

[21] Lyden PD, Hemmen TM, Grotta J, et al. Endovascular therapeutic hypothermia for acute ischemic stroke: ICTuS 2/3 protocol. Int J Stroke 2014; 9: 117–125. 24206528 10.1111/ijs.12151 PMC9673996

[22] Kobeissi H, Ghozy S, Bilgin C, et al. Early neurological improvement as a predictor of outcomes after endovascular thrombectomy for stroke: a systematic review and meta-analysis. J Neurointerv Surg 2023; 15: 547–551. 35636948 10.1136/neurintsurg-2022-019008

[23] Thabane L, Ma J, Chu R, et al. A tutorial on pilot studies: the what, why and how. BMC Med Res Methodol 2010; 10: 1. 20053272 10.1186/1471-2288-10-1 PMC2824145

[24] Harris B, Andrews PJD, Murray GD, et al. Non-invasive head-cooling methods and devices. Health Technol Assess 2012; 16: 1–174. 10.3310/hta16450 PMC4781040 23171713

[25] Abou-Chebl A, Sung G, Barbut D, et al. Local brain temperature reduction through intranasal cooling with the RhinoChill device: preliminary safety data in brain-injured patients. Stroke 2011; 42: 2164–2169. 21680904 10.1161/STROKEAHA.110.613000

[26] Poli S, Purrucker J, Priglinger M, et al. Induction of cooling with a passive head and neck cooling device: effects on brain temperature after stroke: effects on brain temperature after stroke. Stroke 2013; 44: 708–713. 23339959 10.1161/STROKEAHA.112.672923

[27] Kobeissi H, Adusumilli G, Ghozy S, et al. Endovascular thrombectomy for ischemic stroke with large core volume: an updated, post-TESLA systematic review and meta-analysis of the randomized trials. Interv Neuroradiol. Epub ahead of print 28 June 2023. 10.1177/15910199231185738 PMC13305119 37376869

[28] Ferhat S, Bellanger G, Milnerowicz M, et al. Iatrogenic arterial vasospasm during mechanical thrombectomy requiring treatment with intra-arterial nimodipine might be associated with worse outcomes. Eur J Neurol 2024; 31: e16467. 10.1111/ene.16467 PMC11554851 39248014

[29] Pilgram-Pastor SM, Piechowiak EI, Dobrocky T, et al. Stroke thrombectomy complication management. J Neurointerv Surg 2021; 13: 912–917. 34158401 10.1136/neurintsurg-2021-017349 PMC8458081

[30] Buggy DJ, Crossley AW. Thermoregulation, mild perioperative hypothermia and postanaesthetic shivering. Br J Anaesth 2000; 84: 615–628. 10844839 10.1093/bja/84.5.615

[31] Schaller-Paule MA, Foerch C, Bohmann FO, et al. Predicting poststroke pneumonia in patients with anterior large vessel occlusion: a prospective, population-based stroke registry analysis. Front Neurol 2022; 13: 824450. 35250827 10.3389/fneur.2022.824450 PMC8893016

[32] Hill MD, Goyal M, Demchuk AM, et al. Efficacy and safety of nerinetide in acute ischaemic stroke in patients undergoing endovascular thrombectomy without previous thrombolysis (ESCAPE-NEXT): a multicentre, double-blind, randomised controlled trial. Lancet 2025; 405: 560–570. 39955119 10.1016/S0140-6736(25)00194-1

[33] Díaz M, Becker DE. Thermoregulation: physiological and clinical considerations during sedation and general anesthesia. Anesth Prog 2010; 57: 25–32. 20331336 10.2344/0003-3006-57.1.25 PMC2844235

